# Influence of compression therapy following varicose vein surgery: a prospective randomized study

**DOI:** 10.1590/1677-5449.202200522

**Published:** 2023-06-30

**Authors:** Felipe Coelho, Walter Junior Boim Araújo, Sergio Belczak, Eduarda Ferreira Rui, Beatriz Begnini Borsato, Nathália Favaretto Baldesserra, Rodrigo Gomes de Oliveira

**Affiliations:** 1 Pontifícia Universidade Católica do Paraná – PUCPR, Londrina, PR, Brasil.; 2 Universidade Federal do Paraná – UFPR, Curitiba, PR, Brasil.; 3 Centro Universitário São Camilo – CUSC, São Paulo, SP, Brasil.

**Keywords:** venous insufficiency, varicose veins, compression stockings, insuficiência venosa, varizes, meias de compressão

## Abstract

**Background:**

The use of compression dressings after phlebectomy is based solely on clinical experience due to the lack of a unified set of definitive recommendations, which makes clinical practice extremely heterogeneous.

**Objectives:**

To evaluate compression therapy with elastic stockings for 7 days after phlebectomy.

**Methods:**

We randomly allocated 104 lower limbs with disease classified as C1 and C2 to 1 of 2 groups: an intervention group (64 limbs) – wearing elastic compression stockings for the first 7 days after phlebectomy; or a control group (40 limbs) – given conventional bandaging for 24 hours postoperatively. We compared clinical response by analyzing the evolution of symptoms, hematoma formation, and preoperative vs. postoperative limb volume.

**Results:**

Pain (median 1.0 vs. 1.5, p=0.0320) and limb volume (mean 43.7 vs. 99.8, p=0.0071) were significantly improved in patients wearing elastic compression stockings for 7 days after phlebectomy compared with controls.

**Conclusions:**

Use of elastic compression therapy for 7 days after phlebectomy was effective for improving pain and lower limb volume.

## Resumo

**Contexto:**

O uso de curativos após flebectomia é baseado apenas na experiência clínica, visto que não existe um conjunto unificado de recomendações definitivas, o que torna a prática clínica extremamente variável.

**Objetivos:**

Avaliar o uso de terapia elástica compressiva por 7 dias após flebectomia.

**Métodos:**

Cento e quatro membros inferiores, classificados como CEAP C1 e C2, foram randomizados em dois grupos: grupo de intervenção (64 membros) – uso de meia elástica por 24 horas após a cirurgia – e grupo controle (40 membros) – uso de curativo convencional por 7 dias após a cirurgia. A resposta clínica foi comparada por meio de análise da evolução dos sintomas, de hematoma e do volume dos membros antes e depois da cirurgia.

**Resultados:**

Os pacientes submetidos a terapia compressiva elástica apresentaram melhora significativa na dor (mediana 1,0 vs. 1,5; p=0,0320) e no volume dos membros (média 43,7 vs. 99,8; p=0,0071) em comparação ao grupo controle.

**Conclusões:**

O emprego da terapia compressiva elástica por 7 dias após flebectomia mostrou-se efetivo na melhora da dor e do volume dos membros inferiores.

## INTRODUCTION

Compression therapy after sclerotherapy for varicose veins is based on experimental evidence, with proven beneficial effects.^1^ After varicose vein surgery, the use of compression stockings improves visual analogue scale (VAS) pain scores early after surgery,^2^ with no apparent benefit from wearing the stockings for a prolonged period of time.^2,3^

The duration of compression therapy ranges from 2 days to 12 weeks depending on the medical indication and patient compliance is essential for success.^4^ However, in a unified set of recommendations, there is insufficient quality evidence to reach a consensus on use of compression stockings after phlebectomy.^1-14^

Most of the research conducted to date has been limited to comparing different pressures and duration of compression therapy. Due to the variety of therapeutic approaches available, it is necessary to investigate more effective measures and their correct use in the postoperative setting.

The present study aimed to evaluate the impact of compression therapy with elastic stockings for 7 days after phlebectomy for varicose veins, compared with a control group that received leg wrapping with sterile gauze and crepe bandage for 24 hours postoperatively. We evaluated signs and symptoms, hematoma, and volume of the treated limb before and 7 days after the surgical procedure.

## MATERIALS AND METHODS

### Ethical aspects

The study was approved by the institutional Ethics Committee (decision number 3915892), and followed the tenets of the Declaration of Helsinki and Brazilian National Health Council Resolution no. 466/2012. Written informed consent was obtained from each study participant.

### Study design

We conducted a randomized, comparative study of patients undergoing phlebectomy for varicose veins treated with compression therapy with elastic stockings for 7 days postoperatively vs. controls who received leg wrapping with sterile gauze and crepe bandage for 24 hours postoperatively.

### Participants

Participants were recruited from August to October 2019 at a private vein clinic. The study population was a convenience sample of consecutive patients with primary varicose veins of the lower extremities, classified as Clinical, Etiologic, Anatomic, and Pathophysiologic (CEAP) C1 and C2 disease, with indications for surgical treatment with phlebectomy.

Eligible participants were all patients aged 18 years or over undergoing phlebectomy for chronic venous insufficiency of the lower extremities, classified as CEAP C1 and C2 disease. Ultrasound mapping for varicose vein surgery was performed in all patients.

Patients were excluded if they had (1) acute deep vein thrombosis (DVT) or DVT without recanalization on ultrasound imaging, (2) varicose veins with involvement of the great saphenous vein, (3) varicose vein diameters larger than 4mm measured by ultrasound in the standing position, (4) previous documented thrombophilia, (5) cancer (active or in remission), (6) self-reported lung disease, or (7) peripheral arterial insufficiency (ankle-brachial index <0.9).

### Interventions

All patients were evaluated by ultrasound mapping previous to the procedure.

Varicose vein phlebectomies were performed under local tumescent anesthesia. The tumescent anesthesia agent was a 0.1% solution composed by 0.9% saline plus 2% lidocaine, 8.4% sodium bicarbonate and adrenaline. The solution was injected into the areas surrounding all the varicose veins paths to achieve anesthesia. Varicose veins were removed with hooks and clamps, through microincisions made with a number 11 scalpel blade. After local hygiene with saline solution, micropore strips were used to dress the skin incisions.

Using a randomization program, we randomly allocated patients to 1 of 2 groups: an intervention group wearing graduated compression with 20-30 mm Hg thigh-length stockings for the first 7 days after phlebectomy; or a control group given leg wrapping with sterile gauze and 3 units of 3m/20 cm crepe multilayer bandage for 24 hours postoperatively for each treated limb. All patients were treated bilaterally and received the same compression regimen on both lower limbs.

To analyze the impact of compression therapy on the volume of the treated limb, we made 8 measurements of the circumference of the right and left calf at the preoperative visit and on postoperative day 7, and then leg volumes were calculated with the truncated cone mathematical formula.^15^

All patients were asked to complete a questionnaire at the preoperative visit and on postoperative day 7 for analysis of the evolution of signs and symptoms. Patients were asked to use a scale ranging from 0 to 5 (0 = no symptom, 5 = the worse symptom) to indicate levels of pain, tightness, tiredness, burning, edema, and discomfort.

To objectively assess hematoma resulting from the procedure, we photographed the lower limb for subsequent assessment by 2 experts who were blinded to group assignment. The images were acquired in 4 upright views (anterior, right lateral, left lateral, and posterior), and postoperative hematoma formation was scored on a 6-point scale ranging from 0 (no hematoma present) to 5 (severe hematoma).

### Objectives

To determinate the impact of elastic compression therapy for 7 days after phlebectomies for varicose vein treatment vs. leg wrapping with sterile gauze and 3 units of 3m/20 cm crepe multilayer bandage for 24hours on the following signs and symptoms: pain (median -IQR), tightness (mean - SD), tiredness (median -IQR), burning (median -IQR), edema (median - IQR), discomfort (mean – SD), right and left limb volume variation (mean - SD), and assessment of postoperative hematoma formation based on photographic records.

### Statistical analysis

Data were analyzed using MedCalc for Windows, version 9.5.2.0 (MedCalc Software, Mariakerke, Belgium). The sample size was calculated in 100 subjects, employing alpha = 5% and beta = 20% based on pain scores after surgical treatment for varicose veins reported in literature.^1^ We compared differences between the 2 groups using the *t* test for independent samples, for continuous variables with normal distribution, the Mann-Whitney U test, for continuous variables with skewed distribution, or the chi-square test, for categorical variables. We used the Welch test for unequal variances. The Kappa test was used to determinate coefficients for correlations between hematoma assessments by two blinded raters.

We set the level of significance at 5% and calculated power as (1 – β) = 0.8 for all tests.

## RESULTS

Fifty-two patients (50 women and 2 men) and a total of 104 treated lower limbs were randomized. The control group comprised 20 patients (40 limbs) and the elastic compression group comprised 32 patients (64 limbs). There were no statistically significant differences between the groups (Table 1).

**Table 1:** Demographic and clinical characteristics (n=52 patients).

**Variable**	**Elastic compression group (n=32)**	**Conventional bandaging group (n=20)**	**p value**
Female	31 (96.875%)	19 (95%)	0.6898*
Male	1 (3.125%)	1 (5%)	
Age – mean (SD)	44.31 (11.06)	48.20 (14.42)	0.2784†
Standing			
Yes	20 (62.5%)	12 (60%)	0.9103*
No	12	8 (40%)	
Family history			
Yes	25 (78.125%)	16 (80%)	0.8509*
No	7	4	
Disease duration – median (IQR)	7.5 (5.0-10.0)	7.5 (2.5-10.0)	0.4518‡
Physical inactivity			
Yes	25 (78.125%)	16 (50%)	0.8509*
No	7	4	
Previous surgery			
Yes	8 (25%)	4 (12.5%)	0.9378*
No	24	16	
BMI – mean (SD)	26.71 (3.23)	27.54 (3.37)	0.3804^†^
Number of pregnancies – median (IQR)	2 (1-2)	2 (1-2)	0.4780^‡^
Comorbidities	8 (25%)	7 (35%)	0.6457*
Hypertension	2 (6.25%)	3 (15%)	0.5770*
Dyslipidemia	1 (3.125%)	1 (5%)	0.6898*
Depressive disorder	3 (9.375%)	2 (10%)	0.6825*
Anxiety disorder	1 (3.125%)	0 (0%)	0.8107*
Thyroid disease	1 (3.125%)	1 (5%)	0.6898*

SD: standard deviation; IQR: interquartile range; BMI: body mass index.

* Chi-square test.

† *t* test.

‡ Mann-Whitney test.

All patients adhered to the compression regimen prescribed during the first week.

Table 2 summarizes the signs and symptoms assessed preoperatively and 7 days after the procedure. Wearing elastic compression stockings significantly improved pain (p=0.0320). No statistically significant results were obtained for the other symptoms (tightness, tiredness, burning, edema, and discomfort).

**Table 2:** Evolution of signs and symptoms (n=52 patients).

**Variable**	**Elastic compression group (n=32)**	**Conventional bandaging group (n=20)**	**p value**
Pain – median (IQR)	1.0 (0-1)	1.5 (1-2)	0.0320†
Tightness – mean (SD)	1.75 (1.67)	1.50 (1.88)	0.6183*
Tiredness – median (IQR)	1 (0-2.5)	1 (1-3)	0.6859^†^
Burning – median (IQR)	0 (0-0.5)	0 (0-2.0)	0.2323^†^
Edema – median (IQR)	1 (0-1)	0 (0-0.5)	0.1324^†^
Discomfort – mean (SD)	3.66 (2.21)	2.80 (1.79)	0.1513*
Right limb volume variation – mean (SD)	10.33 (51.50)	-31.97 (108.59)	0.1159*
Left limb volume variation – mean (SD)	4.05 (35.97)	-53.44 (91.08)	0.0129*

IQR: interquartile range; SD: standard deviation.

* *t* test.

† Mann-Whitney test.

The volumetric assessment of the lower limbs in the control group showed negative values for both the right (mean, −31.97) and left (mean, −53.44) limbs, indicating that limb volumes were larger after surgery.

In the elastic compression group, limb volume was positive both on the right (mean, 10.33) and left (mean, 4.05) sides. The analysis of postoperative limb volume change showed statistically significant effects in favor of the intervention group (p=0.0071) (Table 2).

Two raters independently scored patients’ images on a 6-point scale (0-5) in 4 views (anterior, right lateral, left lateral, and posterior) to evaluate the impact of compression therapy on postoperative hematoma formation, and the results are shown in Table 3. The results of rater 1 approached significance (p=0.0509) for improvement of hematoma with the use of elastic stockings. The 2 raters’ analyses were considered to be homogeneous, with a correlation coefficient of 0.7640 between them (p<0.0001).

**Table 3:** Impact on postoperative hematoma formation (n=52 patients).

**Score**	**Elastic compression group (n=32)**	**Conventional bandaging group (n=20)**	**p value**
Rater 1 – mean (SD)	2.27 (0.72)	2.74 (0.95)	0.0509*
Rater 2 – mean (SD)	2.42 (0.93)	2.90 (0.90)	0.0744*

Correlation coefficient between blinded raters 1 and 2 = 0.7640 (p<0.0001), Kappa test.

SD: standard deviation.

* *t* test.

## DISCUSSION

Varicose veins of the lower extremities, in their various different manifestations, affect up to 80% of the general population and clinical treatment practice is heterogeneous worldwide.^16^ Compression therapy has a well-established role in conservative treatment and in management of chronic venous insufficiency, with improvements in venous hypertension, leg muscle function, and lower limb venous return. However, international guidelines and current recommendations lack robust evidence to recommend the optimal postoperative compression therapy.^17,18^

The effectiveness of elastic compression after surgical treatment remains unclear. It appears to be useful in preventing DVT and in reducing symptoms such as pain, edema, hematoma, and hemorrhagic complications. However, there is limited evidence on the benefits of compression therapy to improve each specific symptom, in addition to the guidance that must be followed regarding the optimal postoperative compression therapy.^18^

The advantage of water-displacement volumetry is the possibility of direct measurement of objects with irregular shapes. However, the method has problems related to hygiene, is very time-consuming (two successive measurements take about 20-30min), and is not suitable for measuring the volumes of the extremities of patients in the immediate postoperative period. Most studies evaluate edema based on leg diameter variation.

El-Sheikha et al.^12^ and Bakker et al.^19^ reported reduced pain in patients on a compression regimen stipulating continuous wearing of elastic stockings for 1 week, compared with patients wearing stockings only for 2 days. These findings were not confirmed in another study, which found no improvement in pain with the use of compression therapy.^10^ The present study showed a statistically significant improvement in pain (p=0.032) in patients wearing elastic compression stockings vs. controls after 7 days of treatment.

Ye et al.^20^ and Ayo et al.^21^ analyzed postoperative hematoma resulting from phlebectomy between groups receiving short-term vs. long-term compression therapy. Hematoma was assessed using a VAS ranging from 0 (no hematoma present) to 5 (severe hematoma) and a scale based on the anatomic extent of the hematoma. Neither study found any significant difference between groups in terms of hematoma formation. In the present study, the results of rater 1 approached significance (p=0.0509) for improvement of hematoma in the group wearing elastic stockings for 7 days after phlebectomy compared with the control group.

A study conducted to assess edema in the postoperative period after varicose vein surgery found a difference in favor of compression therapy: there was a statistically significant reduction in edema at 14 days compared with the control group.^22^

Data from the present study confirmed a statistically significant difference in limb volume after wearing compression stockings for 7 days compared with the control group (p=0.0071).

The present study has limitations regarding the small sample size and the imbalance between the two groups. Given the fact that there were no statistical differences when the epidemiological data of the sample were analyzed, we assume that the control group achieved its purpose. The fact that the intervention was not blinded between the groups may have influenced the subjects’ perception of pain.

Nevertheless, this finding suggests that 7-day compression therapy in the postoperative period after phlebectomy may prevent edema secondary to the procedure and, consequently, improve patient comfort.

Our study is in agreement with previous publications showing improvement in signs and symptoms regarding the use of compression stockings after phlebectomies, helping vein specialists with management of postoperative patients.

## CONCLUSION

The study concluded that, of the variables studied, use of elastic compression therapy for 7 days after phlebectomy was effective to slightly improve pain scores and achieved a remarkable reduction in lower limb volume. We also showed a potential benefit for improvement of hematoma after compression therapy, as assessed by one of the raters.

Further research is needed to corroborate the findings of the present study and thus provide data that enable development of guidelines for postoperative management of varicose vein surgery.
